# Comparing efficacy of a single intraarticular injection of platelet-rich plasma (PRP) combined with different hyaluronans for knee osteoarthritis: a randomized-controlled clinical trial

**DOI:** 10.1186/s12891-022-05906-5

**Published:** 2022-11-04

**Authors:** Hung-Ya Huang, Chien-Wei Hsu, Guan-Chyun Lin, Huey-Shyan Lin, Yi-Jiun Chou, I-Hsiu Liou, Shu-Fen Sun

**Affiliations:** 1 Department of Physical Medicine and Rehabilitation, Kaohsiung Veterans General Hospital, No 386, Ta-Chung 1st Road, 813 Kaohsiung, Taiwan, Republic of China; 2 Institute of Medical Science and Technology, National Sun Yat-sen University, Kaohsiung, Taiwan, Republic of China; 3 Department of Internal Medicine, Kaohsiung Veterans General Hospital, Kaohsiung, Taiwan, Republic of China; 4 National Yang-Ming University School of Medicine, Taipei, Taiwan; 5 Department of Information Technology and Management, Fooyin University, Kaohsiung, Taiwan, Republic of China; 6 Department of Health-Business Administration, Fooyin University, Kaohsiung, Taiwan; 7 Department of Orthopedic Surgery, Kaohsiung Veterans General Hospital, Kaohsiung, Taiwan

**Keywords:** Hyaluronic acid, Intraarticular injection, Knee osteoarthritis, Platelet-rich plasma

## Abstract

**Background:**

Intraarticular plasma-rich platelet (PRP) and hyaluronic acid (HA) have each been shown to be effective for treating knee osteoarthritis (OA). Evidence supporting the combination therapy is controversial. This study aimed to investigate the efficacy of a single intraarticular PRP injection combined with different HAs in patients with knee OA.

**Methods:**

In this prospective randomized-controlled trial, 99 patients with Kellgren-Lawrence grade 2 knee OA with average knee pain ≥ 30 mm on a 0-100 mm pain visual analog scale (VAS) were randomized into two groups. The PRP + Artz group received a single intraarticular HA (Artz, 2.5 ml, 10 mg/ml) followed by 3 ml PRP (*n* = 50). The PRP + HYAJOINT Plus group received a single intraarticular cross-linked HA (HYAJOINT Plus, 3 ml, 20 mg/ml) followed by 3 ml PRP (*n* = 49). All patients were evaluated before and at 1, 3 and 6 months after injections. The primary outcome was the VAS pain reduction from baseline at 6 months. Secondary outcome measures included Western Ontario and McMaster Universities Osteoarthritis Index (WOMAC), Lequesne index, single leg stance (SLS) test and patient satisfaction.

**Results:**

Ninety-five patients were analyzed by intention-to-treat analysis. Both groups improved significantly in VAS pain, WOMAC, Lequesne index and SLS at 1, 3 and 6 months post intervention (*p* < 0.05). Between-group comparisons showed no significant differences at most follow-up time points, except better improvements in Lequesne index at 1 month (*p* = 0.003) and WOMAC-stiffness score at 6 months (*p* = 0.020) in the PRP + Artz group, and superiority in SLS at 1, 3 and 6 months in the PRP+ HYAJOINT Plus group (*p* < 0.001, *p* = 0.003 and *p* = 0.004). Additional Johnson-Neyman analyses showed that among the patients with baseline WOMAC-pain score > 8.5, WOMAC-function score > 21.7 and WOMAC-total score > 32.0, respectively, those treated with PRP + HYAJOINT Plus injections had better effects in WOMAC-pain, WOMAC-function and WOMAC-total scores than those treated with PRP + Artz at 3 months postinjection (*p* < 0.05). Both groups reported high satisfaction. No serious adverse events occurred during the study.

**Conclusions:**

A single PRP injection combined with Artz or HYAJOINT Plus is effective and safe for 6 months in patients with knee OA. Both injection regimens are potential treatment options for knee OA. Further studies are needed to confirm these results.

**Trial registration:**

The study was registered at ClinicalTrials.gov (NCT04931719), retrospectively. Date of registration 18/6/2021.

**Name of trial registry:**

Comparing efficacy of single PRP combined with different hyaluronans for knee osteoarthritis.

**Level of evidence:**

Therapeutic Level 1.

**Supplementary Information:**

The online version contains supplementary material available at 10.1186/s12891-022-05906-5.

## Background

Knee osteoarthritis (OA) is a common degenerative joint disease that may cause pain, disability and affect patients’ quality of life [1]. Current treatment options include patient education, weight reduction, orthotics, therapeutic exercise, dietary supplement, medication, intraarticular injection of corticosteroid, hyaluronic acid (HA) or platelet-rich plasma (PRP), physical therapies, and surgery [2]. Because articular cartilage has a limited potential to repair, effective therapies remain challenging. Innovative therapy to find the best way for both symptomatic and disease modifying treatment to slow down the progression of OA is an evolving research field. PRP and HA are two commonly utilized intraarticular treatments with disease modifying potential in knee OA [3**–**5]. A new trend of combining these two injections has emerged in recent years.

PRP has anti-nociceptive and anti-inflammatory effects that may reduce pain and modulate the OA process, and it has chondroprotective roles by stimulating endogenous HA production and promoting cartilage matrix synthesis [6–8]. Recent meta-analyses have shown that intraarticular PRP is an effective and safe therapy for knee OA [9, 10]. However, the optimal dose and frequency of PRP use for knee OA remains controversial, ranging from every week to every 3–4 weeks schedule with single to multiple injections [11–13].

Intraarticular HA may promote endogenous HA synthesis, reduce apoptosis rates of chondrocytes, provide joint lubrication and shock absorption, and have chondroprotective effects [14, 15]. Viscosupplementation with HA is effective for knee OA with initial efficacy at 4 weeks, peak effectiveness at 8 weeks and may last for 6 months [16].

PRP combined with HA may benefit from their different mechanisms with some overlap, and may synergistically enhance chondrocyte proliferation, inhibit inflammation, modulate the disease process, and improve joint homeostasis in OA [17–24]. However, there are only very few clinical studies focusing on the combination of PRP and HA for OA [25–30]. Evidence supporting the combined therapy is controversial.

To date, there is no prospective randomized-controlled trial (RCT) investigating the efficacy of a single intraarticular PRP combined with different HA products for treating knee OA. This study aimed to investigate the efficacy of a single intraarticular PRP injection combined with different HAs in patients with knee OA. We hypothesized that intraarticular injection of a single PRP+ Artz reduces pain, similar to a single PRP + HYAJOINT Plus injection, and both injection regimens lead to better functional recovery at 6-month follow-up.

## Methods

### Study design

This prospective, double-blind RCT with 6-month follow-up was conducted in the outpatient rehabilitation department at a university-affiliated tertiary-care medical center from July 2019 until June 2020. The study aimed to compare the efficacy of a single intraarticular PRP followed with different HAs for treating knee OA. Patients were referred from our outpatient orthopedic department with the diagnosis of knee OA. The inclusion and exclusion criteria are shown in Table 1. The study had gained ethical approval from Institutional Review Board of Kaohsiung Veterans General Hospital and followed the Declaration of Helsinki. All participants were instructed about the study and signed an informed consent form. The trial was registered retrospectively at ClinicalTrials.gov (NCT04931719) (date of registration 18/6/2021).

**Table 1:** Inclusion and exclusion criteria

Inclusion criteria	
Aged 20 to 85 years	
Symptomatic knee osteoarthritis with average knee pain ≥ 30 mm on a 0–100 mm pain VAS for more than 6 months despite conservative treatment such as analgesics, NSAIDs, or physical therapy	
Kellgren-Lawrence grade 2 knee osteoarthritis by radiographs taken within previous 6 months	
Willing to discontinue all NSAIDs or other analgesic medication (except for rescue medication) during the study	
No use of physical therapy or changes in orthotic devices during the study	
Radiological evidence of bilateral knee osteoarthritis was accepted if global pain VAS in the contralateral knee< 30 mm.	
Exclusion criteria	
Previous orthopedic surgery on target knee, spine or lower limb	
Disabling osteoarthritis of either hip or foot	
Knee instability, apparent joint effusion or marked valgus/varus deformity	
Known allergy to avian proteins or hyaluronan products	
Pregnant and lactating women	
Intraarticular injections into target knee in the past 6 months	
Therapy with anticoagulants or antiaggregants	
Any specific medical conditions (active infection, rheumatoid arthritis, severe cardiovascular disease, collagen vascular disease, hemiparesis, neoplasm, or hematological disease, etc.), visual, vestibular impairments or poor health status that would interfere with the assessments during the study	

*NSAID* nonsteroidal anti-inflammatory drugs, *VAS* visual analog scale

### Randomization procedures and blinding

The patients were randomized equally into two groups. Randomization was performed by placing group assignments into opaque envelopes by a study coordinator not clinically involved in the trial. Once a patient consented to enter this trial, the person chose one of the envelopes and then was given the allocated injections.

Study blinding was accomplished by having a blinded assessor physician (blinded to the group assignments and not involved in the injection procedure) performed all the assessments. Patients blinding was ensured by avoiding visual access to the injection field with a screen between the patient and his/her flexed knee during the injection process. Additionally, patients were not aware of the group assignments during the study period.

### Intervention

The patients in the PRP + Artz group received one intraarticular injection of Artz (2.5 ml) followed by a single intraarticular injection of PRP (**3** ml). The patients in the PRP + HYAJOINT Plus group received one injection of HYAJOINT Plus (3 ml) followed by a single intraarticular PRP (3 ml) injection. Each patient received 2 different injections, PRP immediately after HA with different needle and syringe. Using aseptic procedures, the injection was performed through anterolateral approach with the knee in 90 degrees flexion by the same experienced physician who did not take part in the assessments or data analysis.

For PRP preparation, 7 mL of venous blood was drawn from each patient’s cubital vein. The blood sample was put into the PLTenus PLUS Platelet Concentrate Separator (TCM Biotech International Corp., Taiwan), which contains acid citrate dextrose as anticoagulant and a specific separator gel that collected platelets and plasma, preventing contamination of leukocytes and red blood cells. After centrifugated for 8 minutes at 500–1200 rpm, the leukocyte-poor PRP (3 mL) was obtained. Evaluated through complete blood count assay, the average platelets concentration is around 463.8 ± 75.4 × 10^3^/ul, which is approximately 2.4 times the baseline platelet concentration.

Artz (Seikagaku Corporation, Japan), an avian-derived HA (2.5 ml, 1% of HA, 10 mg/mL), has a low molecular weight ranging between 620 to 1170 k Daltons (average 900 k Daltons). HYAJOINT Plus (SciVision Biotech Inc., Taiwan), a biological-fermented HA (3 ml, 2% of HA, 20 mg/ml), has been synthesized by chemically crosslinking process by 1, 4-butanediol diglycidyl ether (BDDE) to create an anti-degraded characteristic with a high molecular weight > 15 million Daltons [31].

Demographic and baseline data were collected at baseline. Patients taking analgesics or nonsteroidal anti-inflammatory drugs (NSAIDs) halted them at least 7 days before the baseline evaluation. The patients were prohibited from using analgesics, chondroprotective supplements such as glucosamine or chondroitin, or physiotherapy throughout the study. Acetaminophen, restricted to 4 g/day was permitted as rescue analgesic for knee pain during the study, but it had to be discontinued 24 hours preceding any subsequent visit. Use of rescue analgesic during the study period was documented on a diary card by the patient. Major protocol violations included the initiation of physiotherapy, surgery, and utilization of prohibited drugs. Patients were defined as noncompliant when they missed any follow-up visit.

### Outcome measures

Patients were assessed at baseline visit and at 1, 3, and 6 months after the injections. The primary outcome was the visual analog scale (VAS) pain reduction at 6 months. The patient rated the average pain intensity on knee movement during the past week using a 0–100 mm horizontal VAS line (0 = no pain to 100 = worst pain imaginable) [32].

The secondary outcomes included the Western Ontario and McMaster Universities Osteoarthritis Index (WOMAC, Likert Scale), the Lequesne index, single-leg stance test (SLS), and patient satisfaction (Appendix A) [33–36].

Safety assessments were checked by adverse events reported by the patients during the study and physical findings observed by the evaluator at each follow-up visit. Furthermore, we telephoned the participants at 1 week postinjection to gather information for safety records. A serious adverse event was defined as an event permanently disabling, requiring hospitalization, life threatening or fatal.

### Sample size and statistical analysis

To estimate the required sample size, we used the Statistical Software G*Power 3.1.9.7, based on the statistical method, repeated measures, between-group F-test, and set power = 0.9, alpha = 0.05, number of groups = 2, number of measurements = 4. As there was no preliminary information from literature, Cohen’s effect size f in medium level 0.25 and Cohen’s effect size r of correlation among repeated measures in medium level 0.3 were utilized. The calculated sample size was 41 in each group. With an estimated dropout rate of 20%, 50 subjects per group were planned to be included.

The efficacy data collected were analyzed by intention-to-treat (ITT) analysis. The subjects by ITT analysis contained all participants who received the injection and had at least one post-baseline evaluation. The missing efficacy data in ITT analysis was imputed with the last observation carried forward method.

The Statistical Package for the Social Sciences software version 25.0 (IBM Corp, USA) was used for the statistical analyses. Independent samples t-tests, Fisher’s exact tests, and chi-square tests were performed to compare the baseline characteristics between the two study groups. Repeated measures one-way analysis of variance (ANOVAs) and Bonferroni’s post hoc tests when ANOVAs were significant were performed to explore the changes among baseline, 1, 3, and 6-month follow-ups on primary and secondary outcome measures except for satisfaction within each of the two groups. Differences between the two groups at each follow-up were assessed using independent samples t-test for satisfaction, and independent samples one-way analysis of covariance (ANCOVAs) (using baseline data as the covariates) or Johnson-Neyman techniques for the other outcome measures. We utilized repeated measures one-way ANOVA to examine if there was significant difference in satisfaction among 1, 3, and 6-month follow-ups, and Bonferroni’s post hoc test was used when there was significance. *P* value < 0.05 was considered as statistically significant.

## Results

### Patient characteristics

**Fig. 1:**
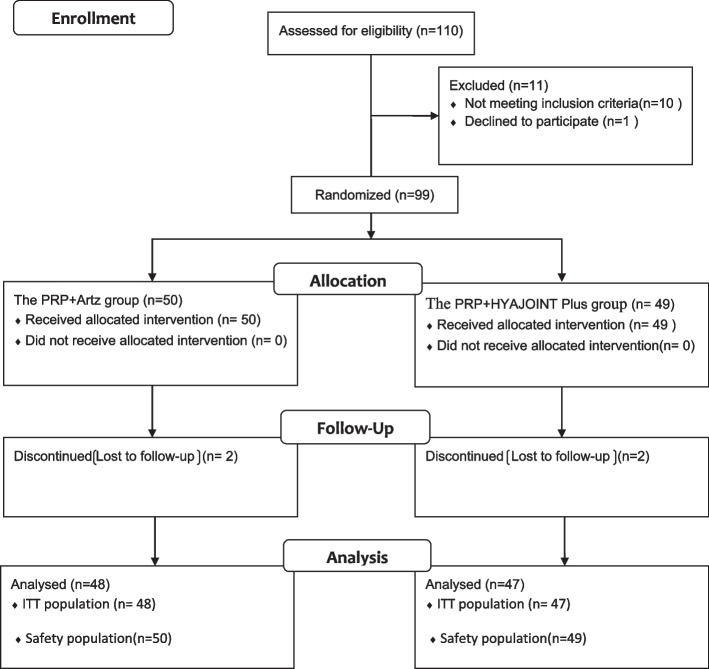
Flow diagram of participants through the trial, ITT = intention-to-treat

Of the 110 screened participants, 10 did not meet the inclusion criteria and one participant declined to take part, thus 99 participants were randomized to either the PRP + Artz group (*n* = 50) or the PRP + HYAJOINT Plus group (*n* = 49) (Fig. 1). Four patients (two in each group) were lost to follow-up, leaving 95 patients available for the ITT analysis at 6 months. We reached the four patients by phone at the time of the missed subsequent visits, and no adverse events were reported. There were no significant differences in baseline characteristics between the 2 groups (*p* > 0.05) (Table 2). The mean age of patients in the PRP ± Artz group (*n* = 48) was 61.9 ± 8.8 years and 64.6% were women, whereas the mean age in the PRP **+** HYAJOINT Plus group (*n* = 47) was 61.0 + 8.1 years and 63.8% were women.

**Table 2:** Demographic and baseline characteristics of the study participants

Characteristic	PRP + Artz (*N* = 48)	PRP + HYAJOINT Plus (*N* = 47)	*P* value
Age, years	61.9 ± 8.8	61.0 ± 8.1	0.590^t^
Gender, female(%)	31 (64.6%)	30 (63.8%)	0.431^a^
Weight, kg	64.0 ± 13.5	67.0 ± 14.0	0.286^t^
Height, cm	161.1 ± 8.6	162.1 ± 7.2	0.522^t^
Body mass index, kg/m^2^	24.5 ± 3.8	25.4 ± 4.4	0.286 ^t^
Employment status (light worker/heavy labor)	40 (83.3%)/8 (16.7%)	41 (87.2%)/6 (12.8%)	0.592^f^
Knee injection side (left/right)	23 (47.9%)/25 (52.1%)	24 (51.1%)/23 (48.9%)	0.759^a^
Disease duration, years	5.3 ± 4.1	5.5 ± 3.3	0.734^t^

The values are given as mean ± the standard deviation or number of patients, with the percentage in parentheses; ^a^χ2-test; ^f^ Fisher’s exact test; ^t^ independent t-test

*P < 0.05

### Primary and secondary outcomes

Significant improvements were observed in both groups in VAS pain, WOMAC (including 3 subscale scores and total scores), Lequesne index scores and SLS among baseline, 1, 3, and 6 months postinjection (Tables 3, 4 and 5).

**Table 3:** Comparisons of primary outcome among groups at different time points and across four waves of times in individual groups

Outcome	PRP + Artz (*N* = 48)	PRP + HYAJOINT Plus(*N* = 47)	A.M.D. (95% C.I.)	*P* value^b^
VAS pain, mm Baseline	42.3 ± 11.2	39.7 ± 10.3	2.6 ^a^ (−4.6, 9.7)	0.483^t^
1 month	23.2 ± 14.7	21.8 ± 15.0	0.9 (−4.5, 8.0)	0.645
3 month	13.1 ± 14.9	11.6 ± 12.4	0.8 (−4.5, 6.0)	0.779
6 month	11.9 ± 16.7	8.7 ± 11.5	2.8 (−3.0, 8.6)	0.347
*P* Value^c^ (post hoc test)	< 0.001* (B > 1 M; B > 3 M; B > 6 M)	< 0.001* (B > 1 M; B > 3 M; B > 6 M; 1 M > 3 M; 1 M > 6 M)		

The values are given as mean ± standard deviation. *VAS* visual analog scale for pain; A.M.D. Adjusted mean difference, *C.I.* confidence interval, B. baseline,^a^M.D. Mean difference, ^b^ Between-group difference determined using independent samples one-way ANCOVA (baseline data as covariate); ^c^Within-group difference determined using repeated measures one-way ANOVA; ^t^ independent t-test; *P < 0.05

**Table 4:** Comparisons of outcomes of WOMAC scales among groups at different time points and across four waves of times in individual groups

Outcome	PRP + Artz (*N* = 48)	PRP + HYAJOINT Plus (*N* = 47)	A.M.D. (95% C.I.)	*P* value^b^
WOMAC-pain Baseline	5.9 ± 3.8	6.0 ± 3.2	−0.1^a^ (−1.5, 1.4)	0.909 ^t^
1 month	3.3 ± 3.1	3.9 ± 2.8	− 0.6 (− 1.6, 0.4)	0.242
3 month	3.4 ± 3.5	3.2 ± 2.1		Fig. 2a (B > 8.5,Plus<Artz)
6 month	3.2 ± 3.2	3.9 ± 2.8	−0.6 (−1.6, 0.4)	0.260
*P* Value^c^ (post hoc test)	< 0.001* (B > 1 M; B > 3 M; B > 6 M)	< 0.001* (B > 1 M;B > 3 M; B > 6 M)		0.599^a^
WOMAC-stiffness Baseline	2.6 ± 1.7	2.3 ± 1.3	0.2^a^ (−0.4, 0.9)	0.424^t^
1 month	1.5 ± 1.4	1.6 ± 1.1	−0.3 (− 0.7, 0.2)	0.307
3 month	1.5 ± 1.6	1.3 ± 1.1	0.05 (−0.4, 0.5)	0.823
6 month	1.3 ± 1.4	1.8 ± 1.1	−0.6 (− 0.1, −1.0)	0.020*
*P* Value^c^ (post hoc test)	< 0.001* (B > 1 M; B > 3 M; B > 6 M)	< 0.001* (B > 1 M; B > 3 M)		
WOMAC-function Baseline	19.7 ± 13.5	21.0 ± 11.9	−1.3^a^ (−6.4, 3.9)	0.634 ^t^
1 month	11.5 ± 10.9	14.2 ± 9.2	−2.2 (−5.7, 1.3)	0.215
3 month	12.6 ± 12.4	10.7 ± 8.5		Fig. 2b (B > 21.7, Plus<Artz)
6 month	11.8 ± 11.2	13.9 ± 11.2	−1.4 (−5.0, 2.2)	0.438
*P* Value^c^ (post hoc test)	< 0.001* (B > 1 M; B > 3 M;B > 6 M)	< 0.001* (B > 1 M; B > 3 M;B > 6 M; 1 M > 3 M)		
WOMAC-total Baseline	28.2 ± 18.4	29.3 ± 15.6	−1.1^a^ (−8.1, 5.9)	0.757 ^t^
1 month	16.2 ± 15.1	19.8 ± 12.3	−3.1 (−7.8, 1.7)	0.204
3 month	17.6 ± 16.9	15.3 ± 11.0		Fig. 2c (B > 32.0, Plus<Artz)
6 month	16.4 ± 15.5	19.5 ± 14.6	−2.5 (−7.4, 2.3)	0.304
*P* Value^c^ (post hoc test)	< 0.001* (B > 1 M; B > 3 M; B > 6 M)	< 0.001* (B > 1 M; B > 3 M; B > 6 M; 1 M > 3 M;6 M > 3 M)		

The values are given as mean ± standard deviation. *WOMAC* The Western Ontario and McMaster Universities Osteoarthritis Index, *A.M.D.* Adjusted mean difference, *C.I.* confidence interval, *B.* baseline, ^a^M.D. Mean difference

**Fig. 2:**
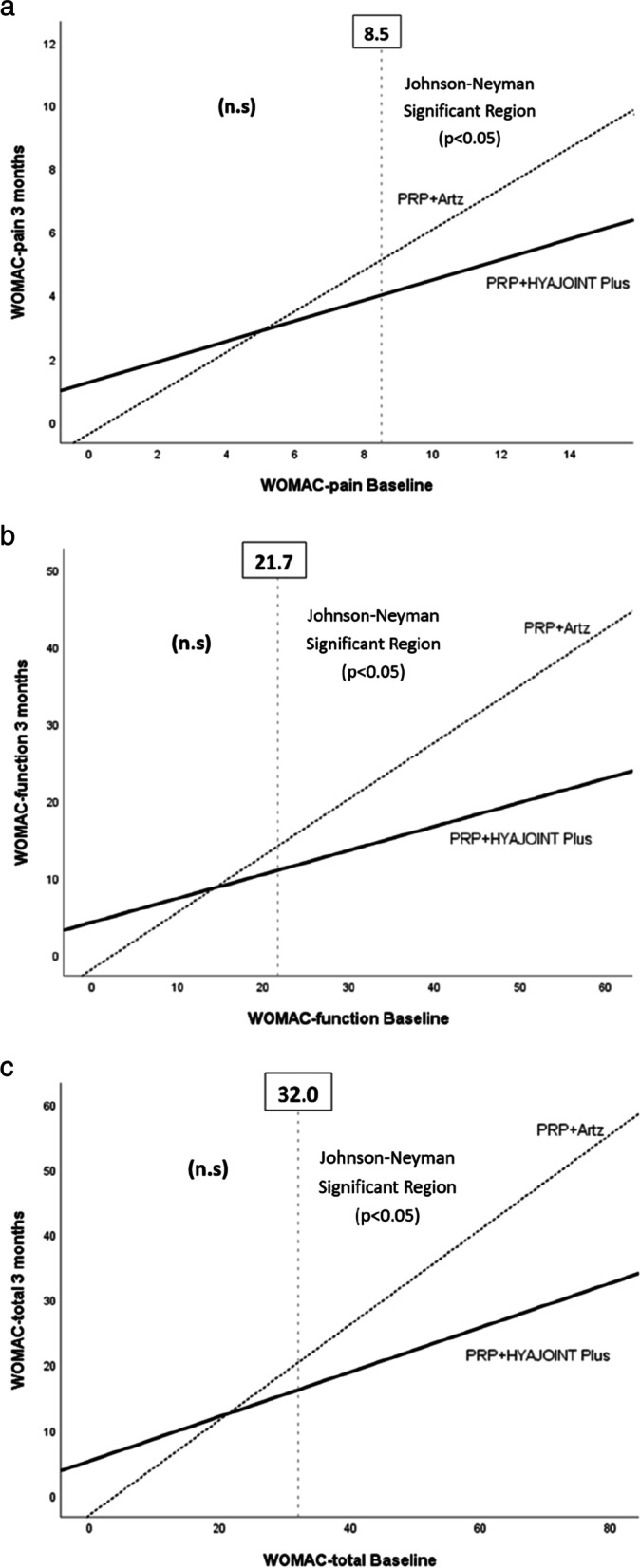
**a**, **b** and **c** Graph showing that, among the patients with baseline WOMAC-pain score > 8.5, WOMAC-function score > 21.7 and WOMAC-total score > 32.0, respectively, those treated with PRP + HYAJOINT Plus injections had superior improvements in WOMAC-pain, WOMAC-function and WOMAC-total scores than those treated with PRP + Artz at 3-month follow-up

^**b**^Between-group difference determined using independent samples one-way ANCOVA (baseline data as covariate) or Johnson-Neyman technique (Fig. 2a, b, and c); ^c^Within-group difference determined using repeated measures one-way ANOVA; ^t^ independent t-test; *P < 0.05

**Table 5:** The comparison of outcome measures of Lequesne index and SLS

	PRP + Artz (*N* = 48)	PRP + HYAJOINT Plus (*N* = 47)	A.M.D. (95% C.I.)	*P* value^b^
Lequesne index Baseline	9.0 ± 3.6	9.1 ± 4.3	− 0.1^a^ (− 1.7, 1.5)	0.897 ^t^
1 month	5.2 ± 3.4	7.4 ± 4.4	−2.1 (−3.5, − 0.8)	0.003^*^
3 month	5.1 ± 3.7	5.9 ± 3.2	−0.8 (−1.9, 0.3)	0.158
6 month	5.0 ± 3.9	5.8 ± 3.8	−0.8 (−2.1, 0.5)	0.240
*P* Value^c^ (post hoc test)	< 0.001^*^ (B > 1 M; B > 3 M;B > 6 M)	< 0.001^*^ (B > 1 M; B > 3 M;B > 6 M; 1 M > 3 M;1 M > 6 M)		
SLS Baseline	31.5 ± 33.8	25.3 ± 21.9	6.2 ^a^ (−5.4, 17.8)	0.293^t^
1 month	35.5 ± 36.8	48.5 ± 44.8	−19.8 (−30.8, − 8.8)	< 0.001*
3 month	39.3 ± 39.4	47.8 ± 35.2	−14.9 (−24.6, − 5.1)	0.003*
6 month	37.4 ± 39.1	44.8 ± 33.9	−13.8 (−23.0, − 4.6)	0.004*
*P* Value^c^ (post hoc test)	0.025^*^ (n.s.)	< 0.001^*^ (B < 1 M;B < 3 M;B < 6 M)		

The values are given as mean ± standard deviation; *SLS* Single limb stance.; *A.M.D.* Adjusted mean difference, *C.I.* confidence interval; *B.* baseline; *n.s.* none significant; ^a^ M.D. Mean difference; ^b^Between-group difference using independent samples one-way ANCOVA (baseline data as covariate); ^c^Within-group difference using repeated measures one-way ANOVA; ^t^ independent t-test; *p value < 0.05

The mean VAS pain in the PRP + Artz group decreased significantly from 42.3 mm at baseline to 23.2 mm, 13.1 mm and 11.9 mm **r**espectively at 1, 3 and 6 months postinjection (Table 3). The PRP + HYAJOINT Plus group showed significant VAS pain reduction from 39.7 mm at baseline to 21.8 mm, 11.6 mm, and 8.7 mm at each follow-up (Table 3). Both groups showed a significant improvement in VAS pain from baseline at 1 month, and the effect remained significant from baseline at 3 and 6 months. Between-group comparisons showed no significant differences at 1, 3 and 6-month postinjection (Table 3).

There were significant improvements in WOMAC scores (including 3 subscale and total scores) from baseline in both groups at 1 month after injection (Table 4). Most of these improvements remained significant from baseline at 3 and 6 months, except that the WOMAC-stiffness score in the PRP + HYAJOINT Plus group remained significant from baseline only till 3 months (Table 4). Between-group comparisons showed no significant differences at any follow-up time-point, except a significant difference in the WOMAC-stiffness score favoring the PRP + Artz group at 6 months (*p* = 0.020) (Table 4).

In the PRP + Artz group, the WOMAC-total scores improved by 31.9% (95% CI, 10.2–53.6%), 38.7% (95% CI, 21.2–56.2%) and 36.5% (95% CI, 14.4–58.6%) from baseline at 1, 3 and 6 months postinjection, whereas the PRP + HYAJOINT Plus group improved by 28.7% (95% CI, 17.1 -40.4%), 46.7% (95% CI, 37.0–56.3%) and 37.4% (95% CI, 26.7–48.1%), respectively. At each follow-up time point, both groups had improvements exceeded the minimum clinically important difference for changes in WOMAC-total scores set at 16% [37].

Additional Johnson-Neyman analyses revealed regions of significant between-group differences in WOMAC-pain, WOMAC-function and WOMAC-total scores at 3 month (Table 4, Fig. 2a, b, c). Among the patients with baseline WOMAC-pain score > 8.5, WOMAC-function score > 21.7 and WOMAC-total score > 32.0, respectively, those treated with PRP + HYAJOINT Plus injections had better improvements in WOMAC-pain, WOMAC-function and WOMAC-total scores than those treated with PRP + Artz at 3 months postinjection (Fig. 2a, b, c).

The Lequesne index scores showed significant improvement in both groups at 1, 3 and 6-month postinjection (*P* < 0.001 and *P* < 0.001, respectively) (Table 5). Between–group comparison showed superior improvement in the PRP + Artz group at 1 month (*P* = 0.003). There were no significant between–group differences at 3 and 6 months postinjection.

The 2 groups improved significantly in SLS during the study (*P* = 0.025 for the PRP + Artz group and *P* < 0.001 for the PRP + HYJOINT Plus group) (Table 5). Between-group comparisons showed superior improvements in SLS for the PRP + HYJOINT Plus group at 1, 3 and 6 months (*P* < 0.001, *P* = 0.003 and *P* = 0.004, respectively) (Table 5).

There was no significant difference between the two groups in patient’s satisfaction (Table 6). The PRP + Artz group reported significantly greater satisfaction at 6 months compared to 1 month. No patients announced dissatisfaction or aggravations of the symptoms compared to preinjection condition during the study.

**Table 6:** Patient satisfaction during the study

Satisfaction	PRP + Artz (*N* = 48)	PRP + HYAJOINT Plus (*N* = 47)	M.D. (95% C.I.)	*P* value^a^
1 month	71.0 ± 19.7	72.0 ± 18.5	−1.0 (− 8.8, 6.8)	0.804
3 month	76.4 ± 18.9	74.6 ± 14.3	1.8 (−5.0, 8.6)	0.601
6 month	77.9 ± 15.1	71.9 ± 18.6	6.0 (−0.9, 12.9)	0.087
*P* value^b^ (post hoc test)	0.019^*^ (1 M < 6 M)	0.229 (n.s.)		

The values are given as mean ± the standard deviation. *M.D.* Mean difference, *C.I.* confidence interval; n.s. none significant

^a^ Between-group difference using independent t-test

^b^Within-group difference using repeated measure one-way ANOVA; *p value < 0.05

Rescue analgesic consumption decreased significantly in both groups. The demand for rescue analgesic (acetaminophen) fell from an average of 15.3 ± 6.1 tablets weekly at baseline to 7.4 ± 2.4, 6.6 ± 2.7 and 7.1 ± 2.3 tablets weekly at 1, 3 and 6-month follow-up in the PRP ± Artz group (*p* < 0.001 compared with baseline), whereas the analgesics consumption fell from 14.8 ± 6.7 tablets weekly at baseline to 7.1 ± 2.6, 6.2 ± 2.8 and 6.8 ± 2.5 tablets weekly respectively in the PRP+ HYAJOINT Plus group (*p* < 0.001 compared with baseline). There were no significant between-group differences.

### Safety outcomes

Ninety-nine patients who received the injections were included in the safety population. The incidence of adverse events was similar in both groups. Six patients (12.0%) in the PRP+ Artz group and 7 patients (14.3%) in the PRP + HYAJOINT Plus group reported transient knee pain and swelling after injection. The symptoms were mild and resolved uneventfully within 2 days, requiring no analgesics. No serious adverse events happened throughout the investigation period. Adverse events did not result in study discontinuation in the 2 groups.

## Discussion

This was the first prospective double-blind RCT designed to compare the efficacy of a single PRP combined with different molecular-weight HA for treating patients with Kellgren-Lawrence grade 2 knee OA. The study revealed that both injection regimens provided pain reduction and functional improvement with high patients’ satisfaction and no serious adverse events within the 6-month follow-up period.

The primary goal for intraarticular knee injection is pain reduction. In the PRP + Artz group, the mean VAS pain scores reduced by 67.5% (95% confidence interval (CI), 50.7–84.3%) from baseline at 6 months, whereas the PRP + HYAJOINT Plus group had VAS pain reduction by 77.0% (95% CI, 67.8–86.2%), respectively. Reaching this level of pain reduction suggests an achievement in chronic pain clinical trials, using the criteria that pain reduction of at least 30% indicates at least moderate clinically important differences [38]. The results are similar to our recently published research with a mean VAS pain reduction of 79.5% from baseline after a single PRP combined with single HYAJOINT Plus injections at 6 months for treating knee OA [28]. Previous meta-analysis reported that HA reduces knee pain by 40–50% compared with baseline levels [39]. In our previously work, patients with Kellgren-Lawrence grade 2 or 3 knee OA had a mean VAS pain reduction of 56.2% from baseline at 6 months after a single intraarticular HYAJOINT Plus injection [31]. Li et al. found a median VAS pain reduction of 33.3% from baseline at 6 months after a single intraarticular PRP in patients with Kellgren-Lawrence grade 1 or 2 knee OA [40]. Although it is not possible to directly compare these studies, the apparently greater VAS pain reduction (67.5 and 77.0%, respectively) in our study population at 6 months may indicate that PRP combined with HA could be advantageous for treating knee OA.

Both groups showed significant improvements in all WOMAC subscale scores and total scores through the 6-month period, supporting the efficacy of combined PRP + HA in relieving pain, stiffness and improving function for patients with knee OA. A score improvement by 30–40% at the follow-up assessment was defined by Lequesne as an effective form of treatment [41]. In this study, the mean Lequesne index scores improved by 4.0 points (44.4% improvement from baseline) in the PRP + Artz group at 6 months, compared with 3.3 points (36.3% improvement from baseline) in the PRP + HYAJOINT group. The 2 groups met the criteria mentioned above to determine treatment effectiveness.

This was the first clinical trial comparing SLS after PRP combined with different HAs for knee OA. SLS test is an objective test of static balance. Control of standing balance is a crucial indicator of physical functioning and hazard of falling in the knee OA population [42]. OA may cause pain, reduce muscle strength and impair lower limb proprioception, which may lead to low activity level with exacerbation of instability, thus may hinder effective and timely reactions to keep balance [43]. The two novel combined-injection therapies resulted in improvement of not only pain and function, but also static balance function. Interestingly, we found that the PRP + HYAJOINT Plus group exhibited significantly better performance in SLS than the PRP + Artz group at each follow-up (Table 5). Further studies to explore the effects of knee OA on balance and the impacts of PRP + HA on the joint may help clarify the possible mechanisms of disability, and may allow more successful treatment of knee OA.

Several in vitro studies and preclinical studies provided the rationale for the application of PRP + HA [17–24]. Anitua et al. had shown that combined PRP + HA could significantly improve cell mobility and lead to better regenerative capability compared to PRP alone [21]. Marmotti et al. found that adding HA to PRP could effectively improve the osteochondral repair [22]. PRP + HA might cooperatively activate surface receptors, trigger release of signaling molecules and finally enhance chondrogenesis in human articular chondrocyte [17]. PRP + HA might stimulate cellular growth and help restore the articular extracellular matrix [24]. Moreover, HA might act as a scaffold for cartilage repair and as a carrier for the adhesion of stem cells [44].

Clinical studies evaluating OA patients treated with PRP + HA are limited [20, 25–30]. In two retrospective studies comparing 3 weekly injections of PRP + HA versus PRP alone in patients with knee OA, the authors concluded that combination therapy was not superior [20, 27]. Dallari et al. compared PRP, HA and PRP + HA in their hip RCT, and they reported that PRP + HA did not offer additional benefit over PRP at 2, 6 and 12 months [30]. In contrast, we recently showed that a single PRP + HYAJOINT Plus achieved better VAS pain reduction than PRP alone at 6 months for treating knee OA [28]. Lana et al. compared the efficacy of 3 injections of PRP, HA and PRP + HA biweekly for patients with knee OA, they found that PRP + HA showed better outcomes than HA alone at 1 year, and superior function at 1 and 3 months than PRP alone [26]. Yu et al. reported that PRP + HA significantly reduce arthralgia of knee OA and improved the histological parameters compared with PRP or HA alone [29]. It is worthy to note that Lana et al. used two patient-reported outcomes (VAS and WOMAC) and Yu et al. used only one (WOMAC) scale [26, 29]. Our study relied on several different assessments, including objective measurement of static balance and patients’ global satisfaction. Recent meta-analysis by Zhao et al. showed that PRP + HA had greater benefit for pain and function in patients with knee OA, compared to PRP alone [45]. Superior benefits were confirmed in another meta-analysis by Karasavvidis et al., who reported that PRP + HA had better clinical results over HA monotherapy [46]. The longest follow-up period in the two meta-analyses was 1 year only, thus the long-term efficacy of combined PRP + HA for chronic pathologies such as OA remained unknown.

Previous researches regarding PRP or HA in knee OA showed that patients with lower degrees of cartilage degeneration achieved superior outcomes as opposed to those affected by advanced OA [9, 47]. In this study, we included patients with Kellgren-Lawrence grade 2 OA only, as patients with moderate or end-stage knee OA might have lower levels of pain relief and may be diluting study results if included in the treatment cohort [47]. The ideal candidates for PRP + HA injections have never been reported. Interestingly, we found that among patients with baseline WOMAC-pain score > 8.5, WOMAC-function score > 21.7 and WOMAC-total score > 32.0 respectively, those treated with PRP + HYAJOINT Plus injections had superior improvements in WOMAC-pain, WOMAC-function and WOMAC-total scores than those treated with PRP + Artz at 3 months postinjection. Previous researches had shown that PRP + HA provided benefits in elderly patients with advanced knee OA, suggesting that the combination therapy could offer a chance for patients with severe OA not eligible for knee replacement to delay, or prevent the need for surgery [48, 49].

HA products are different in molecular weight, concentration, source (animal or fermentation), volume and structure (linear or cross-linked), dosage (number of injections) thus making them heterogeneous with different efficacy and safety. Previous meta-analysis revealed that intraarticular higher molecular weight HA (≥3000 kDa) had superior efficacy and safety than did those of lower molecular weight (< 1500 kDa); biological fermented-HA exhibited significantly fewer injection site flare-up and knee effusion than did avian-derived HA [50]. In our study, PRP combined with two different HAs showed similar efficacy and safety at 6 months, with some discrepancy between them at different time points. The complex interaction between PRP, HA and the articular milieu are important determinants of therapeutic outcomes. The ideal HA to combine with PRP has yet to be defined.

The adverse events for intraarticular PRP and HA were most commonly reported as minor acute flares that resolved within days, which were very similar to the adverse events seen in our study [51]. Zhao et al. reported that the incidence of adverse events did not differ between PRP + HA and PRP or HA alone, indicating that the safety of the three treatment regimens is similar [45].

Several limitations existed in the study. First, this was a single center study targeting patients with Kellgren & Lawrence grade 2 knee OA only. The results were not applicable to all the OA populations with different radiographic severity. Second, the sample size was small and the follow-up time was short. Larger studies with longer follow-ups are recommended to explore the long-term efficacy. Third, we did not have a single PRP, single HA, a saline injection or a sham control group for comparison, thus it is hard to know the relative contribution of each therapy to the functional gains. The improvements might suggest possible placebo effects, as recent meta-analysis highlight that the high and long-lasting placebo effects of knee injections should not be overlooked [52]. Fourth, the injection volume was different between the 2 groups. The higher injected volume in the PRP+ HYAJOINT Plus group might cause a dilution of growth factors or excessive distension of joint capsule that could affect the outcomes. Fifth, the study protocol was approved after study termination. Further research with high-resolution magnetic resonance imaging or histological study for the evaluation of cartilage and joint structure may possibly provide more objective data in the efficacy evaluation. The cost-effectiveness, the ideal number, volume and frequency of injections, the potential use in joints other than the knees, as well as the role for repeated series of injections must be clarified in the future.

## Conclusions

This study provided evidence that both injection regimens of PRP + Artz and PRP + HYAJOINT Plus were effective and safe for 6 months in patients with symptomatic knee OA. The PRP + Artz group showed better improvements in Lequesne index at 1 month and WOMAC-stiffness score at 6 months, whereas the PRP+ HYAJOINT group revealed superiority in SLS at 1, 3 and 6 months. We recommended more high-quality researches to help confirm the results.

## Supplementary Information

**Additional file 1.**

## Data Availability

The datasets used and/or analyzed during the current study are available from the corresponding author on reasonable request.

## Abbreviations

PRP: Plasma-rich platelet
HA: Hyaluronic acid
OA: Osteoarthritis
VAS: Visual analog scale
WOMAC: Western Ontario and McMaster Universities Osteoarthritis Index
SLS: Single leg stance test
RCT: Randomized-controlled trial
CONSORT: the Consolidated Standards of Reporting Trials
NSAID: Nonsteroidal anti-inflammatory drugs
CI: Confidence interval
MCID: Minimum clinically important difference
