# Supplementary material for: Comparing efficacy of a single intraarticular injection of platelet-rich plasma (PRP) combined with different hyaluronans for knee osteoarthritis: a randomized-controlled clinical trial
Source: BMC Musculoskelet Disord. 2022 Nov 4;23:954. doi: 10.1186/s12891-022-05906-5 (PMC9635114; doi:10.1186/s12891-022-05906-5)
Supplement: Supplementary file 1 — Additional file 1. [file 12891_2022_5906_MOESM1_ESM.doc]

**Appendix A**

Description of the secondary outcome measures:

The Western Ontario and McMaster Universities Osteoarthritis Index (WOMAC, Likert Scale) is a self-administered survey containing 24 items divided into 3 subscales measuring pain (5 items, score range 0–20), stiffness (2 items, score range 0–8), and physical function (17 items, score range 0–68). The maximal score is 96. Higher scores represent worse conditions.

Lequesne index is used to assess severity of knee symptoms during the last week. It is validated and includes 5 questions pertaining to pain or discomfort, 2 questions dealing with maximum distance walked, and 4 questions about activities of daily living. The maximal score is 24. Higher scores indicate worse function.

Single-leg stance test (SLS) is done by asking the participant to stand on the study limb without contacting the free limb to the ground or performing excessive trunk or upper body movements for as long as possible. Each participant performed 3 attempts, and the maximum time among the attempts is recorded.

Patients were asked to rate their treatment satisfaction compared to the preinjection condition, utilizing a 100 mm VAS (0= completely dissatisfied, 100=completely satisfied).
